# Evaluation of a student clinical research education program in addiction medicine

**DOI:** 10.1080/07853890.2022.2154833

**Published:** 2023-01-11

**Authors:** Jules Canfield, Ve Truong, Agata Bereznicka, Carly Bridden, Jane Liebschutz, Daniel P. Alford, Richard Saitz, Jeffrey H. Samet, Alexander Y. Walley, Karsten Lunze

**Affiliations:** a Section of General Internal Medicine, Clinical Addiction Research and Education Unit, Boston Medical Center, Boston, MA, USA; b University of Pittsburgh School of Medicine, Pittsburgh, PA, USA; c Boston University Aram V. Chobanian & Edward Avedisian School of Medicine, Boston, MA, USA; d Department of Community Health Sciences, Boston University School of Public Health, Boston, MA, USA; e Opioid Overdose Prevention Pilot Program, Massachusetts Department of Public Health, Boston, MA, USA

**Keywords:** Addiction medicine, medical education, MSSRP, experiential learning, research training

## Abstract

**Objective:**

To evaluate an experiential student clinical addiction research program by analyzing its components, evaluation survey data, and scientific outputs.

**Methods:**

In 1995, we established a summer research program supporting trainees to gain exposure to clinical addiction research careers. This curriculum employed a three-pronged approach that combined mentored research training, didactic education, and clinical observerships for medical students and other trainees to acquire experience with addiction medicine and research. Utilizing the Kirkpatrick model as program evaluation framework, we analyzed evaluation data from programmatic surveys (didactic seminar evaluations, overall program surveys) and conducted qualitative feedback exploration.

**Results:**

Between 2007 and 2019, 56 trainees and 26 faculty mentors participated in the curriculum. To date, 25 students published 38 papers with their faculty mentor. Analysis of the past 12 years of program evaluation data demonstrated that students highly valued individually-mentored research experiences. They indicated that seminars familiarized them with the foundations of different clinical care models and career trajectories in addiction medicine. Clinical observerships provided students with patient contacts in various multidisciplinary addiction treatment settings. These experiences, perhaps most importantly hearing about patients’ lived experiences, meaningfully informed various research and didactic activities.

**Conclusions:**

This summer student research program successfully introduced students to addiction medicine and research, manifested by high peer-reviewed publication productivity. While our program engaged and involved committed mentors and inspired mentees to pursue professional paths in addiction research, it did not specifically incorporate attention to equity and diversity into program planning and implementation. Going forward, the program will improve equity by increasing the recruitment of trainees from disadvantaged groups and engaging underrepresented faculty.

**KEY MESSAGES**
- Summer programs can be effective in engaging medical students and trainees in research early in their trajectory and inspire them to incorporate research into their careers.
- Programs that integrate experiential addiction research learning, i.e. mentored research activities, didactic sessions, and clinical observerships, can provide trainees with a profound understanding of substance use disorder treatment and research.

## Introduction

Substance use disorders are an ongoing societal, economic, and public health challenge in the United States. Over 20 million adults in the U.S. have a substance use disorder [1]. In 2021, deaths due to drug overdose rose to over 100,000, an increase of 28.5% from the 78,000 deaths the prior year [2]. Over the past decade, rates of overdose deaths have increased among all age groups [3]. Substance use disorders are also estimated to cost the U.S. $442 billion every year—from healthcare-related expenses to decreased work productivity [4].

Medical education and research training preparing physicians to address the substance use crisis have been insufficient to meet needs [5–7], and this in part explains why only one in 10 Americans with a substance use disorder are in treatment [8]. Little training time devoted to substance use disorders, a lack of experienced faculty, minimum support from institutional administrators, and few clinical and training sites have been barriers for medical programs to implement addiction-specific curricula [5]. A history of negative provider attitudes towards patients with substance use disorders has led to fewer diagnoses and poorer treatment outcomes for these individuals [6]. Sufficient numbers of adequately trained clinician scientists and other researchers are crucial to improve the diagnostic, screening, and treatment approaches for individuals with substance use disorders. Implementation of clinical research training programs that involved medical students has demonstrated improved research productivity and interest in these learners’ careers [9–12]. However, little guidance exists on developing and implementing addiction medicine research programs for students.

In response, we developed an addiction summer research program combining research training, seminar sessions, and clinical observations at the Boston University Aram V. Chobanian & Edward Avedisian School of Medicine and Boston Medical Center for medical, undergraduate, and graduate students to introduce them to the field of addiction research and clinical care as a career pathway.

To inform our evaluation methodology, we performed a systematic search of manuscripts describing medical student research programs, to understand how other programs were implemented and evaluated, and how our program may contribute to the literature and understanding of similar programs. PubMed searches using the terms ‘summer research program’ and ‘medical student research program’ yielded 53 papers published from 1960 to 2021, which reported a range of research training, from summer research programs to longitudinal experiences to combined MS or MD/PhD degrees. We focused on the 17 references specifically detailing summer research programs in the U.S. and Canada, 59% of which (*n* = 10) described research productivity in the form of manuscripts and/or abstracts published by students, 35% (*n* = 6) assessed student interest in research as a future element of their career, and 35% (*n* = 6) discussed increases in student knowledge and skills involving research. Some 30% of papers (*n* = 5) also assessed student satisfaction with or perceived value of the program itself. Our evaluation expands the literature surrounding summer student research programs as it focuses specifically on an experiential addiction research education program.

The aim of this study was to evaluate our experiential student clinical addiction research education program by analyzing its scientific outputs, components, and evaluation survey data. Informed by our systematic literature search, we further aimed to synthesize insights, lessons learned, and participant feedback gained over the years. We included quantitative and qualitative measures of these outcomes in our evaluation. We also assessed the effectiveness of our program against the Kirkpatrick model, a well-known framework for training evaluation.

## Methods

Funded by the National Institute on Drug Abuse (NIDA) since 2001, the *Clinical Addiction Research and Education* (CARE) Program at Boston Medical Center (teaching hospital for the Boston University Aram V. Chobanian & Edward Avedisian School of Medicine) offers first year medical students interested in an addiction medicine a research curriculum over the 8 weeks of their summer term break. Over the past several years, we have extended participation to other trainees, including undergraduate and graduate students. Trainees typically identify our summer program through our website [13] or referral from faculty members and collaborators. We engage participants in a three-pronged summer research program:

1. **Research training**: Students can become involved with a variety of activities in the clinical research realm with their assigned faculty mentor: literature reviews, participant recruitment and interviewing, data collection, data entry, data analysis, and manuscript and abstract presentation. Faculty and their research staff orient students and assign them activities.
2. **Didactic education**: Eight weekly seminars led by CARE Unit faculty (i.e. physician-researchers and PhD-level scientist experts focused on addiction medicine) provide a foundation in addiction research. At the beginning of each seminar, faculty discuss how they became involved in addiction medicine and their career trajectory. The spectrum of addiction medicine topics covered in these seminars is listed in Table 1.
3. **Clinical observerships**: According to students’ expressed interest, they engage in multiple observer experiences to gain exposure to models of addiction care delivery. These observations expose students to clinicians and patients’ perspectives on addiction care including the screening, diagnosis, and treatment of substance use disorders. The various clinical observation options (Table 2) complement and enhance trainees’ participation in the seminars and their research activities [13]. They provide students with insights into the operation of clinical programs and the interactions between patients and clinicians.

**Table 1.** Topics of 2008–2019 CARE summer research program seminars.*

Seminar title	Times presented
Addiction and International Research	2
Addiction Research Using Large Databases	4
Alcohol: A Public Health Perspective	3
Alcohol, Drugs & HIV: Clinical Research Insights	3
Alcohol, Other Drugs, and Health: Current Evidence Newsletter	1
Building your Career in Addiction Research	3
Contingency Management and Stimulants	1
Harm Reduction	1
Hepatitis C Virus Infection in Injection Drug Users	2
How to Read Research Articles	2
Maintaining an Interest in Addiction Medicine in Your Medical Education	1
MassHEAL—A Proposal to Reduce Community Overdose by 40% in 3 Years	1
Medication Treatment for Addictive Disorders	3
Opioids: The Good, The Bad, and the Ugly/Opioid Use Disorders	9
Overdose Crisis in Massachusetts	4
Overdose Prevention and Naloxone Distribution	4
Screening and Brief Intervention for Alcohol Use Disorder	10
Smoking Cessation & Health Disparities	1
Social Determinants of Addiction	3
Substance Abuse and Homelessness in Boston	4
Substance Use Policy Research	3
Substance Use Disorders as a Developmental Disorder	2
Trauma and Substance Abuse	4

*There were no seminar sessions held in 2010 or 2013.

**Table 2.** 2015–2019 Summer trainee experiences at observation sites.*

Observation site	Number of trainees observing at site	Mean score for accommodation of host at the observation site (1.0–5.0 scale, with 1.0 being least accommodating *vs.* 5.0 as being most accommodating)
Addiction Consult Service Treatment service at Boston Medical Center for inpatients with substance use disorders	21	4.81
Buprenorphine Medical Appointment Program Weekly group visit focused on buprenorphine treatment, patient education, and peer support	7	5.0
CATALYST Clinic Clinic for adolescents and young adults with substance use disorders	10	4.91
ECHO Addiction treatment training for health centres that utilizes videoconferencing technology	5	4.80
Faster Paths Clinic Urgent care center that refers patients with substance use disorders to treatment services	10	4.63
FAST PATH Clinic Clinic providing addiction treatment and HIV primary care	20	5.0
FAST PATH Support Group Weekly group for FAST PATH patients focused on providing support and facilitating recovery	19	4.63
Hope House Residential treatment program for adult men with substance use disorders	18	4.67
Opioid Treatment Program Clinic for methadone-maintained patients who receive dose adjustments and medical care	28	4.79
Office-Based Addiction Treatment (OBAT) Clinic Clinic where nurse care managers provide medications for the treatment of substance use disorders and counselling	16	4.63
Primary Care Clinic Clinic where selected physicians treat patients with substance use disorders for primary care	10	4.50
Woods-Mullen Shelter Physician-led tour of a local housing facility for people experiencing homelessness	7	5.0
Overall, how useful was the observation series to you?	33 trainee responses	4.85 average (1.0–5.0 scale, with 1.0 being less useful *vs.* 5.0 being most useful)
How clear were directions and scheduling for your observations?	33 trainee responses	4.48 average (1.0–5.0 scale, with 1.0 being less clear and 5.0 being most clear)

*Observation sites with <5 trainees observing were not included in the table.

### Application process

After a review of student curricula vitae and a brief description of their research interests and learning goals, we match the students to ongoing research projects and clinical observation experiences with faculty and available projects. We accepted most students including those who were not matched with a mentor for a supervised research project. All students from our institution received a stipend, as did most students from other institutions.

### Evaluation data sources

Since 2008, we have collected evaluation data from participants following each weekly seminar, and since 2015 after each clinical observation by emailing a link to a trackable online questionnaire using the Research Electronic Data Capture (REDCap). The questionnaires assessed learners’ perceptions of the most useful ideas and concepts from the seminar and clinical experience as well as suggestions for improvements or modifications. We also measured the overall experience and quality of clinical observations at program end, using quantitative scales and open-ended questions. We conducted a thematic analysis as a descriptive approach to free-text responses to our evaluation surveys [14]. Beginning with open coding, using descriptive codes for a single round of coding, we coded emerging themes, organizing them into several categories or patterns such as satisfaction, inspiration, engagement, and involvement. We also conducted a search of peer-reviewed publications that program participants have authored with their summer faculty mentor. We searched PubMed for authors using participant and mentor names for the time period 2007–2019 program and compiled specifically those publications that participants had published with their summer faculty mentor following their respective Medical Student Summer Research Program (MSSRP) period (Table 3).

**Table 3.** Publications by 2007–2019 CARE MSSRP Participants with their faculty mentor.

Publication	Year published	CARE MSSRP participant(s)	CARE faculty mentor(s)
Are opioid dependence and methadone maintenance treatment (MMT) documented in the medical record? A patient safety issue	2009	Farrar	Walley, Samet, Alford
The impact of a 25-cent-per-drink alcohol tax increase	2012	Daley	Naimi
Methadone dose, take home status, and hospital admission among methadone maintenance patients	2012	Filippell	Walley
Buprenorphine treatment for hospitalized, opioid-dependent patients: a randomized clinical trial	2014	Dossabhoy	Liebschutz
A new scale of the U.S. alcohol policy environment and its relationship to binge drinking	2014	Nguyen	Naimi
Adherence to prescription opioid monitoring guidelines among residents and attending physicians in the primary care setting	2015	Dossabhoy	Liebschutz
Differential risk factors for HIV drug and sex risk-taking among non-treatment-seeking hospitalized injection drug users	2015	Dossabhoy	Liebschutz
Alcohol policies and impaired driving in the United States: Effects of driving- *vs.* drinking-oriented policies	2015	Nguyen	Naimi
Youth drinking in the United States: Relationships with alcohol policies and adult drinking	2015	Nguyen	Naimi
The relationship between alcohol taxes and binge drinking: evaluating new tax measures incorporating multiple tax and beverage types	2015	Nguyen	Naimi
Hepatitis C virus testing and treatment among persons receiving buprenorphine in an office-based program for opioid use disorders	2016	Carey	Tsui
Who would pay for state alcohol tax increases in the United States?	2016	Daley	Naimi
Prescribe to prevent: Overdose prevention and naloxone rescue kits for prescribers and pharmacists	2016	Lim	Walley
Long-term retention in office based opioid treatment with buprenorphine	2017	Hui, Kim	Weinstein
Psychoactive medications and disengagement from Office Based Opioid Treatment (OBOT) with buprenorphine	2017	Hui, Kim	Weinstein
Very early disengagement and subsequent re-engagement in primary care Office Based Opioid Treatment (OBOT) with buprenorphine	2017	Hui, Kim	Weinstein
What do providers want to know about opioid prescribing? A qualitative analysis of their questions	2017	Hodgkin	Cushman
Polypharmacy and risk of non-fatal overdose for patients with HIV infection and substance dependence	2017	Lerner, Mauricio	Walley
Tapering off and returning to buprenorphine maintenance in a primary care Office Based Addiction Treatment (OBAT) program	2018	Hui, Kim	Weinstein
Communication between nurse care managers and patients who take opioids for chronic pain: Strategies for exploring aberrant behavior	2018	Husain	Liebschutz
Polypharmacy and risk of falls and fractures for patients with HIV infection and substance dependence	2018	Lerner, Mauricio	Walley
Which patients receive an addiction consult? A preliminary analysis of the INREACH (INpatient REadmission post-Addiction Consult Help) study	2019	D’Amico	Weinstein
Reasons for opioid discontinuation and unintended consequences following opioid discontinuation within the TOPCARE trial	2019	Husain	Liebschutz
Factors associated with help seeking by community responders trained in overdose prevention and naloxone administration in Massachusetts	2019	Lim	Walley
Alcohol, age, and mortality: Estimating selection bias due to premature death	2019	Stadtmueller	Naimi
Process evaluation of counselling delivered by a patient navigator in an efficacious smoking cessation intervention among low-income primary care patients	2019	Ulrich	Lasser
Inpatient addiction consultation and post-discharge 30-day acute care utilization	2020	D’Amico	Weinstein
Stigma and quality of co-located care for HIV-positive people in addiction treatment in Ukraine: a cross-sectional study	2020	Dutta	Lunze
TB stigma and its correlates among HIV-positive people who inject drugs in Ukraine	2020	Dutta	Lunze
Resilience and diabetes self-management among African-American men receiving primary care at an urban safety-net hospital: a cross-sectional survey	2020	Jenkins	Lasser
Characteristics and receipt of medication treatment among young adults who experience a nonfatal opioid-related overdose	2020	Patel	Bagley
Trends in cannabis involvement and risk of alcohol involvement in motor vehicle crash fatalities in the United States, 2000‒2018	2021	Buczek	Naimi
Emergency department utilization among people living with HIV on chronic opioid therapy	2021	Kulkarni	Thakarar, Walley
Shorter outpatient wait-times for buprenorphine are associated with linkage to care post-hospital discharge	2021	Price	Roy, Walley
Rejection of patients with opioid use disorder referred for post-acute medical care before and after an anti-discrimination settlement in Massachusetts	2021	Rosenmoss	Kimmel
Competing risks of women and men who use fentanyl: ‘The number one thing I worry about would be my safety and number two would be overdose’	2021	Sampath	Gunn
Age-based preferences for risk communication in the fentanyl era: 'A lot of people keep seeing other people die and that’s not enough for them’	2021	Sampath	Gunn
Violence, HIV risks, and polysubstance use among HIV-positive people who inject drugs in Ukraine	2021	Schoenberger	Liebschutz

### Evaluation framework

The Kirkpatrick model [15] and outputs we commonly found in the literature review guided our evaluation approach. The Kirkpatrick model has four levels of training and education evaluation: Reaction, Learning, Behavior, and Results. For the assessment of participant reaction to the program in level 1 of the Kirkpatrick model, we documented participants’ satisfaction with program components. We also shared level 1 satisfaction data with the didactic speakers, which would allow them to modify their teaching in future program iterations. For level 2 of the model assessing learning and its impacts on skills, we documented useful concepts that students self-reported learning during the seminars as well as whether or not their overall learning goals were achieved during the program and why. Our level 3 analysis of behaviors that reflect how participants utilized their learning provides summary statistics for learners’ output of peer-reviewed, PubMed-indexed research papers published with their program mentor. Our evaluation did not include a level 4 analysis, which has a primary focus on organizational results or outcomes: as program graduates continued their practice in various organizations, we deemed systemic program impacts beyond the scope of our evaluation.

## Results

### Overview of program/participants

Between 2007 and 2019, 56 trainees and 26 faculty mentors participated in the CARE summer research program. Of those, 37 were Boston University medical students and five were medical students from other universities. On average, five students participate in the program each year. We did not document participant and faculty demographics during this time period. Examples of research projects that students completed included understanding post-partum changes in methadone dose among women in methadone maintenance treatment, assessing hepatitis C testing and treatment in an office-based program for opioid dependence treatment, and characterizing stigma related to both HIV and substance use disorder.

Students participated in 7–8 weekly seminars led by CARE faculty. Since 2008, there were over two dozen addiction medicine topics presented, including addiction and its treatment, substance use screening and brief interventions, and social determinants of addiction. The majority of presenters were addiction medicine clinical researchers and education scholars whose talks were rooted in the development and widespread usage of evidence within addiction care. They shared with trainees their career trajectory and how they became involved in addiction medicine and/or research, and many discussed within these seminars their own research studies and associated results.

### Level 1: Satisfaction with seminar evaluation and clinical observerships

Students indicated that before the program, they had little information about the topics presented in the seminars (Table 1) and thought that they would not have been exposed to addiction topics in their medical curriculum otherwise. One medical student summarized how the seminars encouraged them to critically question existing evidence: ‘A useful takeaway message was that even research results that have been widely accepted and adopted by authorities, [it] can be misinformed or change over time. We must always be willing to re-evaluate and reconsider research findings and the interventions they support’. Another participant suggested aligning the seminars closer to students’ advocacy aspirations and ‘to incorporate into the didactic sessions more discussion about the racial/ethnic and class disparities that exist in substance use disorder treatment, the ways in which drug criminalization policies have disproportionately impacted communities of colour, and the role of addiction medicine physicians in advocating for more equitable programs and policies’.

Trainees appreciated faculty’s discussion about their career paths and backgrounds, as this medical student expressed: ‘I enjoyed listening to an interesting story along a career path. I’m not 100% sure what I want to do with my life right now, and it was great to hear that it doesn’t matter. [*This faculty*] changed her mind plenty of times in her life, and she clearly ended up very successful’.

Trainees generally rated observation experiences highly (Table 2). Most participated in 4–5 observations on average during their time in the program. Qualitative feedback indicated that the clinical observations helped students relate their research to individuals with substance use disorders. Trainees appreciated that hosts were welcoming and ‘open and willing to teach and explain…’, that observations ‘really furthered my learning this summer, and the experiences I had will stick with me for a long time’. While some observation sites (e.g. FAST PATH Clinic, Buprenorphine Medical Appointment Program) were rated more highly than others host accommodation-wise, the qualitative feedback from students did not yield specific indications of reasons for the higher ratings.

Trainees identified the following as their most useful or valuable experiences: (1) the high quality of faculty mentorship while working on their research project, (2) drawing inspiration from CARE faculty career paths during the seminar series, and (3) relating their research to patient experiences through narratives about addiction and recovery they heard during observations. Students welcomed the interrelated program components and ‘the well-organized framework of research experience, didactic sessions, and clinical observations’. Participants particularly valued the exposure to people with lived experiences: ‘My most valuable experience was meeting patients with substance use disorders who were in various stages of recovery. They came from diverse backgrounds and had different stories, and I feel very privileged to have had the opportunity to learn from them this summer’.

### Level 2: Reported skills and attitudes

Trainees summarized that they gained a solid understanding of addiction medicine and started developing research skills. Didactic sessions allowed them with the opportunity to learn more about the different facets of substance use disorders. In seminar evaluations, students were asked to list the most useful concept or idea presented to them in a particular didactic. The majority of trainees listed at least one new piece of information gained from each seminar—whether that was understanding the difference between methadone and buprenorphine, recognizing how social determinants of health affect substance use disorders, or diving more deeply into various study designs in addiction research and their respective strengths and weaknesses.

Students were also asked in the REDCap survey if their learning goals going into the program were met or unmet by the end of the curriculum; Overwhelmingly, they stated their goals had been achieved, with variations on which components were most effective in doing so. One trainee stated: ‘I wanted to learn about the basics of conducting good research and how to present it. I gained great experience by being a part of the summer research training program, was able to design an analytic plan and present it’. Another student remarked: ‘My goals this summer were mostly research-based goals, but in terms of care, my ultimate goal was to get to the root of why I wanted to be a doctor. And at the end of this summer, I feel that I am well on the journey of realizing this goal through the hours spent doing hands-on research as well as participating in observations’.

Finally, students felt inspired by the program’s mission of serving the underserved: ‘I have learned so much from the CARE faculty, research staff, and patients. It was exciting to be among a group of people so passionate about caring for some of the most vulnerable people in our community. These patients are survivors and I admire the strength it takes to work towards recovery’.

### Level 3: Program outputs

There are several initiatives and impacts that have evolved from this program: (1) Program graduates founded the *Boston University Addiction Education Coalition* [16], a student interest group to raise awareness about substance use disorders and their treatment by healthcare professionals. They organize seminar sessions and educational workshops for medical students, liaise with student outreach groups serving Boston’s disadvantaged communities, and partner with our summer program to provide its members with the opportunity to participate; and (2) the Addiction Medicine Rotation for primary care residents, a week-long, immersive rotation of clinical experiences was modelled after this program’s observations. Finally, 25 students have authored 38 peer-reviewed publications with their summer faculty mentor to date (Table 3).

## Discussion

This program evaluation of our summer addiction research program for medical students revealed that it familiarized students with research methods and populations, and data from the willingness to pursue addiction research suggests that the program inspired participants to careers in addiction research. Consistent with existing literature, several of our trainees indicated an increased interest in medical research as a career path and many noted high satisfaction with our programmatic components, particularly with regard to observation and didactic experiences [10,11,17–20]. We found programmatic satisfaction and perceived educational value to be equally high among our summer trainees compared to other program evaluations that assessed this metric [17,19,21–23]. Through our triad of research mentoring, observation experiences, and didactic seminars, trainees have stated increased knowledge about addiction, consistent with the several papers in our literature review that noted knowledge increases in the respective topic areas [17,20,24,25]. Finally, several students in this program have published their research with their faculty mentor as manuscripts in peer-reviewed journals. The more than 1.5 papers produced on average by learners in this program compare favourably with the 1 or 2 papers published by mostly longer, more intense curricula published in the literature [9,10,17,18,22,23,26–28].

While some experiential summer programs have provided medical students with research training [9–12], this program complements existing ones by its focus on addiction science [29]. It also complements existing American Society of Addiction Medicine and International Society of Addiction Medicine workshops and other activities for students, which can build skills and professional identity as an addiction science researcher, but typically do not result in publications.

### Limitations and future directions

Heretofore, increasing trainees from underrepresented groups in science and medicine has not been an explicit goal in our programming despite its importance in improving equity in addiction care. We added this focus in 2020 by collecting demographic information (e.g. race, ethnicity, sexual orientation, gender identity, trainee status) from program trainees to form a database on which to measure current and future progress. Additional initiatives include expanding the learner community beyond medical students to include other trainees such as international and graduate students to create a diverse, mutually beneficial learning environment. Some research faculty had undergraduate participants in NIDA Summer Research Training program for underrepresented minorities who were included in the seminars [30]. Promoting our program to student and national organizations of underrepresented groups will allow us to reach out to a wider pool of potential applicants. Additionally, we will expand our focus on identifying diverse faculty mentors, critical for long-term sustainability in addressing educational and health disparities, who are underrepresented in medicine (URiM) and from various disciplines. We will actively encourage these faculty members to share their career trajectories, as students have found this aspect of seminars both revealing of possibilities and as inspiring to pursue a career in addiction medicine. We also have incorporated a didactic that focuses on diversity, equity, and inclusion in the addiction workforce and the history of racism and addiction.

There are several other changes we plan to incorporate moving forward based on what we have learned. We will henceforth conduct long-term systematic follow-up with participants to document their research involvement and outputs several years after their participation in the program. We now distribute pre- and post-surveys to our participants, to measure and compare how research attitudes, knowledge, and interests evolve over the program.

Future iterations could broaden our current program scope. Volunteer projects, social media campaigns and advocacy- or policy-based projects by students and their mentors could complement the existing experiential clinical research methods training. Further development and broadening of the current program’s scope and targeting learners from disadvantaged populations could help expand its reach and foster the next generation of addiction researchers to address the national opioid crisis and the global substance use challenge.

## Conclusions

Experiential summer research programs such as the one reported here are effective in engaging medical students and trainees in research early in their trajectory and in inspiring them for research careers. Programs that integrate experiential addiction research learning (i.e. mentored research activities, didactic sessions, and clinical observerships) can provide trainees with a profound understanding of substance use disorder treatment and research; and with individuals with lived experience that this program aims to serve.

## Data Availability

The datasets used and/or analyzed for the development of this manuscript are available from the corresponding author on reasonable request.
